# One-year monitorization of the gut colonization by multidrug resistant bacteria in elderly of a single long-term care facility

**DOI:** 10.1093/jacamr/dlaf008

**Published:** 2025-02-08

**Authors:** Cristina Colmenarejo, Concepción Rodríguez-Jiménez, Francisco Javier Navarro, Ana Belén Mateo, Eva María Pellejero, Rosa María Belda-Moreno, Roberto Ureña-Méndez, Raúl Pérez-Serrano, Soledad Illescas, José Ramón Muñoz-Rodríguez, Rosa del Campo

**Affiliations:** Servicio de Microbiología, Hospital General Universitario de Ciudad Real, Ciudad Real, Spain; Servicio de Microbiología, Hospital Universitario Ramón y Cajal, and Instituto Ramón y Cajal de Investigación Sanitaria (IRYCIS), Madrid, Spain; Long-term care facility Gregorio Marañón, Ciudad Real, Spain; Long-term care facility Gregorio Marañón, Ciudad Real, Spain; Long-term care facility Gregorio Marañón, Ciudad Real, Spain; Long-term care facility Gregorio Marañón, Ciudad Real, Spain; Long-term care facility Gregorio Marañón, Ciudad Real, Spain; Servicio de Farmacia, Hospital General Universitario de Ciudad Real, Ciudad Real, Spain; Servicio de Microbiología, Hospital General Universitario de Ciudad Real, Ciudad Real, Spain; Departmento de Microbiología, Facultad de Medicina, Universidad de Castilla-La Mancha, Ciudad Real, Spain; Traslational Research Unit, Hospital General Universitario de Ciudad Real, Ciudad Real, Spain; Servicio de Microbiología, Hospital Universitario Ramón y Cajal, and Instituto Ramón y Cajal de Investigación Sanitaria (IRYCIS), Madrid, Spain; CIBER de Enfermedades Infecciosas, Instituto de Salud Carlos III, Madrid, Spain; Facultad de Ciencias de la Salud, Universidad Alfonso X El Sabio, Villanueva de la Cañada, Madrid, Spain

## Abstract

**Objective:**

To monitor the gut colonization by multidrug resistant (MDR) bacteria in residents of a single long-term care facility (LTCF) in relation to their clinical evolution, antibiotic consumption and mortality risk.

**Methods:**

In a total of 187 voluntarily enrolled residents, five rectal swabs samples were recovered over 1 year. Selective media were used to isolate MDR bacteria. Clinical data related to infections, antibiotic consumption and mortality were recovered. Mortality risk among residents who were MDR colonized and non-colonized was compared by Kaplan–Meier curves.

**Results:**

Globally, 25% of residents have gut colonization by ESBL-producing *Escherichia coli* with a lack of other pathogens such as *Acinetobacter baumannii* or *Clostridioides difficile*. Monitoring of ESBL-producing *E. coli* colonization for 1 year allowed to us to establish three categories among residents: 48.6% never colonized, 15.5% had a persistent colonization, and the remaining 35.8% presented intermittent colonization. The rates of mortality, infections and antibiotic exposure were comparable among ESBL-producing *E. coli* colonized and non-colonized residents, except for the intermittent colonization group in which a higher and statistically significant mortality rate was observed. As expected, urinary and respiratory tract infections were the most prevalent infectious pathologies in the LTCF, with amoxicillin/clavulanate and fluoroquinolones being the most prescribed antibiotics. A high percentage of ESBL-producing *E. coli* (28%), and fluoroquinolone resistance were detected in clinical samples.

**Conclusions:**

The monitoring of gut colonization by MDR microorganisms in a single LTCF for 1 year demonstrated the predominance of ESBL-producing *E. coli*. Almost half of the residents were resistant to its colonization, whereas in 15.5% of them gut colonization was stable. Incidence of infectious episodes and antibiotic exposure were comparable between colonized and non-colonized subjects, but the group with the highest risk of mortality was that with intermittent colonization by ESBL-producing *E. coli*.

## Introduction

Gut colonization by multidrug resistant (MDR) microorganisms including extended spectrum beta lactamase (ESBL)-producing Enterobacteriaceae is not an infrequent event in environments apart from hospitals; in fact, a global spread into the community has been documented with current prevalence rates of 17% of faecal colonization in healthy individuals.^1^ There are no local data on gut colonization prevalence in healthy people in the community, but according to EARSS (https://www.ecdc.europa.eu/sites/default/files/documents/AER-antimicrobial-resistance.pdf), the ESBL rates in clinical samples of our country are 10% to 25% for *Escherichia coli*, and 25% to 50% for *Klebsiella*.

Gut colonization is a complex event influenced by various human factors such as induction of host immune response, as well as the protection exerted by the gut microbiota itself,^2^ mainly due to different competitive interactions leading to niche exclusion, among others. Colonization is usually an asymptomatic and silent process until antibiotic exposure occurs; in fact, the first stage in many systemic infections, mainly sepsis, is due to an intestinal overgrowth of microorganisms usually following antibiotic consumption.^3^

The elderly are particularly vulnerable to gut colonization by MDR bacteria, as they experience frequent hospital admissions and often receive multiple antibiotic treatments.^4^ Moreover, they are at higher risk for sepsis due, among other factors, to a depleted gut microbiota because of the ageing process. This situation contributes to an increasing propensity towards MDR bacterial colonization/infection events with the frequent involvement of *Clostridioides difficile*, which is associated with significant morbidity and mortality rates.

As a result of the increase in life expectancy, a significant proportion of elderly reside in long-term care facilities (LTCFs). The environmental epidemiology of these centres is a complex scenario that coincides with the frequent introduction of community microorganisms carried by newly admitted residents, but also by those acquired in tertiary hospitals to where this population is frequently referred. In fact, antimicrobial stewardship in LTCFs is strongly recommended,^5,6^ although implementation is difficult in these settings. In a previous study, we described the high prevalence of gut colonization by ESBL-producing *E. coli* in a single LTCF.^7^ Although the dynamic of gut colonization by MDR bacteria in LTCFs has been explored in other studies, including in our country,^8,9^ scarce data are still available, and conclusions cannot easily be unified as each centre has its own special features. The aim of the present study was to describe the dynamics of intestinal colonization by MDR bacteria in 187 participants enrolled in a single LTCF over 1 year, and to relate it to the characteristics of their infections, antimicrobial resistance rates and antibiotic consumption.

## Methods

The study was conducted between July 2018 and July 2019 in a single LTCF located in Ciudad Real, Spain, with the approval of the ethics committee of the General University Hospital of Ciudad Real (assigned code C-155). Participation in the study was offered to the entire centre, although only 187 residents confirmed their participation, and those or their legal representatives signed a written consent form. Residents usually did not leave the centre, although they received visits from their relatives.

Faecal carriage of *C.s difficile*, MDR-*Acinetobacter baumannii* and ESBL-producing Enterobacterales was determined along the year of survey through five rectal swab samples in July and October of 2018 and in January, April and July of 2019. Swabs were immediately seeded on a Brilliance ESBL Agar (Thermo Scientific, Basingstoke, UK), CLO agar (bioMérieux) and LEEDS *Acinetobacter* medium (bioMérieux), and plates were incubated at 37°C for 48 h, under aerobic conditions, except for the CLO agar plates that were incubated in anaerobiosis. The growing colonies were identified by MALDI-TOF spectrometry (Bruker Daltonik, Bremen, Germany). ESBL production was confirmed by the double-disc synergy test.

The microbiological data related to clinical samples and antibiotic consumption from the entire LTCF, were retrieved from the database of the Microbiology and Pharmacy Services of the General University Hospital referred to before. Hospital admissions, clinical data and diagnosed infections (whether caused by MDR, antibiotic treatments and surgical procedures) were recorded.

Stata and R software (v.4.4.1) programs were used for statistical analysis. Qualitative variables are expressed as counts and frequencies, *n* (%). Quantitative variables are presented as means and standard deviations (M ± SD). To evaluate patient survival time as a function of colonization category, a Kaplan–Meier test was performed by factoring by the groups described in Table 4 (continued alive and intermittent 1, 2 and 3 sampling sessions)

## Results

### Gut colonization by MDR bacteria in the 187 participants

Considering the five sampling sessions, a total of 824 rectal swabs were collected from the 187 initial participants, however, 46 of them (24.5%) died during the study year (10 in the second sampling, eight in the third, 19 in the fourth and nine in the fifth). MDR-*A. baumannii* and *C. difficile* colonization was detected in three residents without clinical symptoms (Table 1). ESBL-producing *E. coli* was detected in 216 (26.2%) samples from 91 residents and ESBL-producing *Klebsiella* in 16 (1.9%) samples from four residents. Coexistence of both ESBL-producers’ microorganisms in the same person was not observed.

**Table 1.** Sequential distribution of the major findings along the study

	July 2018 (*n* = 187)	October 2018 (*n* = 177)	January 2018 (*n* = 169)	April 2019 (*n* = 150)	July 2019 (*n* = 141)
Faecal colonization	(*n* = 187)	(*n* = 177)	(*n* = 169)	(*n* = 150)	(*n* = 187)
ESBL-*E. coli*	55 (29.4)	50 (28.2)	33 (19.5)	36 (24.0)	42 (29.7)
ESBL-*K. pneumoniae*	4 (2.1)	3 (1.6)	4 (2.3)	4 (2.6)	0
*A. baumannii*	2 (1.0)	3 (1.6)	3 (1.7)	1 (0.6)	1 (0.7)
*C. difficile*	0	1 (0.5)	2 (1.1)	3 (2.0)	3 (2.1)
Infectious episodes	118 (63.1)	58 (32.7)	84 (49.7)	63 (42.2)	41 (29.0)
Respiratory tract	78 (41.7)	36 (20.3)	55 (32.5)	38 (25.6)	16 (11.3)
Urinary tract	34 (18.1)	22 (12.4)	28 (16.5)	16 (10.8)	20 (14.1)
Skin and soft tissues	15 (8.0)	10 (5.6)	12 (7.1)	10 (6.7)	4 (2.8)
Antibiotic therapy	101 (54.0)	59 (33.3)	83 (49.4)	61 (41.2)	40 (28.3)
Amoxicillin/clavulanate	17 (9.0)	20 (11.3)	28 (16.6)	21 (14.2)	9 (6.4)
Cephalosporin 3rd generation	31 (14.4)	12 (6.7)	16 (9.4)	2 (1.2)	5 (3.5)
Fluoroquinolones	51 (27.2)	10 (5.6)	28 (16.6)	26 (17.6)	9 (6.4)
Fosfomycin	19 (10.1)	13 (7.3)	15 (8.9)	10 (6.8)	8 (5.7)
Aminoglycosides	4 (2.1)	1 (0.5)	2 (1.1)	0	0
Azithromycin	19 (10.1)	2 (1.1)	5 (2.9)	0	0
Cotrimoxazole	9 (4.8)	9 (5.1)	7 (4.1)	1 (0.6)	3 (2–1)
Others	10 (5.3)	11 (6.2)	9 (5.3)	8 (5.4)	8 (5.7)
Infections by MDR	8 (4.2)	4 (2.2)	2 (1.1)	0	2 (1.4)
Hospital admissions	17 (9.0)	8 (4.5)	12 (7.1)	4 (2.7)	2 (1.4)
Surgical procedures	16 (8.5)	3 (1.6)	4 (2.3)	2 (1.3)	2 (1.4)

**Figure 1.**
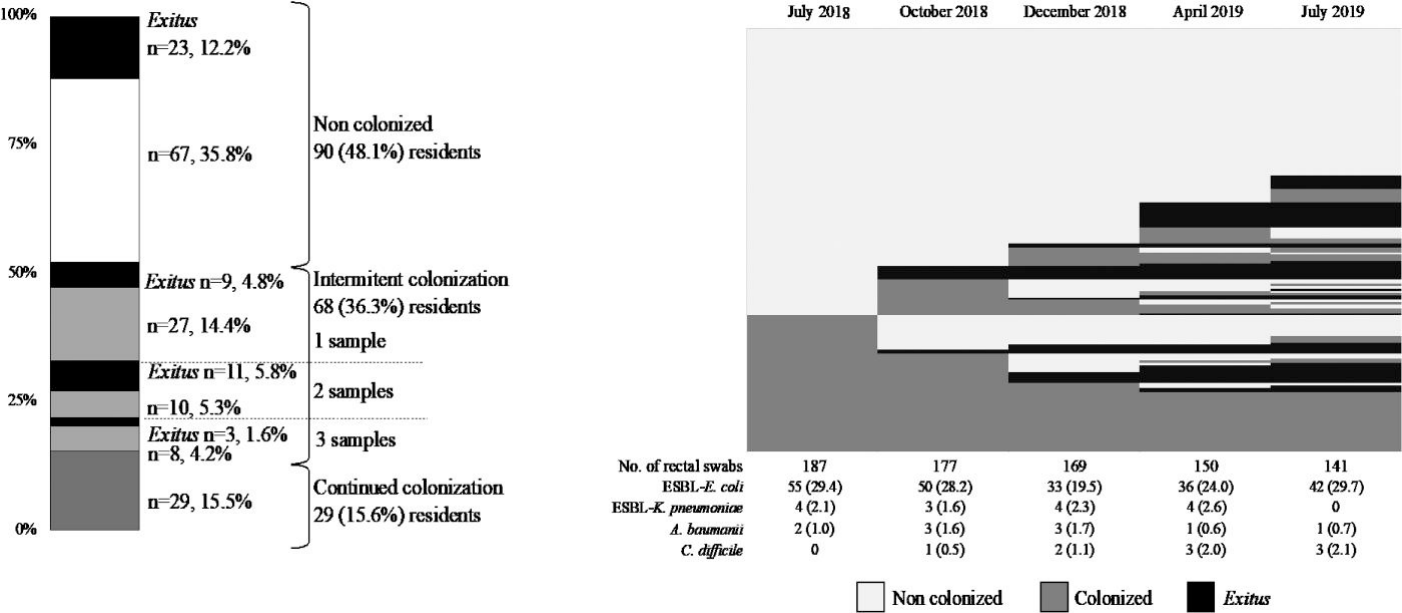
(a): Global classification of the 187 residents according to their ESBL-producing *E. coli* gut colonization and their survival condition, as well as their distribution in the five sampling sessionss. (b): Dynamics of patients’ colonization statuses during sampling. Upper, patients who were never colonized. Lower, patients with continuous colonization. Centre, the evolution of those patients with intermittent colonization, also including deaths. (c) Number and percentage of MDR microorganisms detected in each sampling.

The positive ESBL-producing microorganism detection in one out of the five samples was considered intermittent colonization, while on the other hand, when the ESBL-producers isolates were found in four out of five samples, the negative result was attributed to a probable low load of sample, this was considered a false negative result and ascribed to established colonization in all five samples. The spatial distributions of samples and colonization results are shown in Figure 1, defining three categories: 48.6% as non-colonized, 15.5% as continued colonization and finally, 35.8% as intermittent colonization (Table 2). It is important to note that in the non-colonized category, 65 patients who remained alive during the five samplings and 26 residents who died without ESBL-colonization were grouped together; but in the continued colonization group, only those residents who were alive during the five samplings were included. Finally, we detected intermittent colonization (more than two negative samples after one positive sample) in 32 residents and 20 participants had a single ESBL detection, not detecting any differential features in both groups.

**Table 2.** Characteristic of the different groups according to their gut colonization by ESBL-producing *E. coli*

MDR gut colonization	Median age (range)	Length of stay at LTCF	Barthel index median ± SD	Infections in the six previous months (%)	Diaper/ urinary catheter	UTIr	DM	*Exitus*
Non-colonized-alive (*n* = 67/187, 35.8%)								
Male (*n* = 20, 29.8%)	83.7 (64–100)	4 y (3 m–11 y)	58.2 ± 30.8	11 (55.0%)	10/1	2	5	
Female (*n* = 47, 70.1%)	85.2 (64–96)	5 y (3 m–13 y)	42.1 ± 26.6	27 (57.4%)	23/0	5	19	
Non-colonized *exitus* (*n* = 23, 12.2%)								
Male (*n* = 6, 26.0%)	80.0 (62–93)	4 y (1 y–7 y)	56.6 ± 30.9	5 (83.3%)	0/0	0	2	6/26 (23.0%)
Female (*n* = 17, 73.9%)	85.9 (51–98)	4 y (1 m–13 y)	25.3 ± 30.7	12 (70.5%)	7/0	3	9	17/64 (26.5%)
Continued alive (*n* = 29/187, 15.5%)								
Male (*n* = 10, 34.4%)	80.8 (63–92)	3 y (5 m–6 y)	47.0 ± 31.5	7 (70%)	5/1	2	3	
Female (*n* = 19, 65.5%)	88.5 (73–97)	4 y (2 m–13 y)	31.5 ± 25.0	14 (73.6%)	9/0	5	2	
Intermittent 1 sampling (*n* = 36/187, 19.2%)								
Male (*n* = 13, 36.1%)	83.3 (51–95)	7 y (4 m–20 y)	63.4 ± 29.7	5 (38.4%)	5/0	1	3	3/13 (23.0%)
Female (*n* = 23, 53.1%)	82.7 (67–91)	3 y (2 m–6 y)	43.6 ± 30.7	16 (69.5%)	13/1	2	8	6/8 (26.0%)
Intermittent 2 samplings (*n* = 21/187, 11.2%)								
Male (*n* = 8, 38.0%)	86.1 (75–97)	4 y (2 m–8 y)	34.2 ± 30.8	6 (75.0%)	5/1	2	0	4/8 (50.0%)
Female (*n* = 13, 61.9%)	84.9 (70–93)	6 y (1 m–15 y)	23.4 ± 31.02	8 (61.5%)	9/0	4	5	7/13 (53.8%)
Intermittent 3 samplings (*n* = 11/187, 5.8%)								
Male (*n* = 7, 63.6%)	83.8 (77–91)	4 y (1 m–9 y)	49.2 ± 33.5	5 (71.4%)	3/0	1	3	2/7 (28.5%)
Female (*n* = 4, 36.3)	86.5 (83–92)	4 y (3 m–6 y)	12.5 ± 5.0	3 (75.0%)	4/0	1	2	1/4 (25.0%)

### Current infection status in the LTCF

During the year of the study, the hospital received a total of 242 samples for microbiological analysis from the LTCF; urine (47.5%) wound exudate (33.9%) and ulcer exudate (16.9%), while the remaining origins including sputum, faeces and others were scarce (1.8%). The percentage of the isolated microorganisms and their antimicrobial resistance patterns are shown in Tables 3 and 4, and antibiotic consumption during the period of study is shown in Table 5. Although respiratory tract infections were the most frequently observed, no samples were sent for microbiological analysis, as is usually recommended in mild processes. As expected, *E. coli* and *Proteus mirabilis* were the most frequent microorganisms isolated from urinary tract infections. The high percentages of *Staphylococcus aureus* and *Pseudomonas aeruginosa* from wound and ulcer exudates should also be noted. The prevalence of ESBL-producing *E. coli* and methicillin resistance in *S. aureus* (MRSA) was higher than expected, as well as the rate of fluoroquinolone resistance in all microorganisms.

**Table 3.** Distribution of microorganisms informed in the 242 clinical samples from the whole LTCF residents that were sent to the hospital during the study year

	Percentage of microorganisms by sample (%)				
Microorganism	Urine	Urinary catheter	Wound exudate	Ulcer exudate	Others
*Escherichia coli*	55.8	50.0	15.0	6.2	
*Proteus mirabilis*	15.4		12.5	12.5	
*Klebsiella pneumoniae*	9.6	25.0		6.2	33.3
*Enterococcus faecalis*	0.8			6.2	
*Pseudomonas aeruginosa*	3.8		7.5	18.7	
*Providencia stuartii*	3.8	25.0			
*Staphylococcus aureus*	1.9		32.5	31.2	
*Klebsiella oxytoca*	1.9				
*Enterobacter cloacae*	1.9				
*Corynebacterium striatum*			12.5	6.2	
*Morganella morganii*			10.0	3.2	
*Staphylococcus epidermidis*			5.0	3.2	
*Citrobacter freundii*			2.5		
*Acinetobacter baumannii*			2.5	1.4	33.3
*Candida albicans*				3.2	
*Streptococcus pneumoniae*					33.3
Polymicrobial	2.0				
No. of samples (%)	106 (43.8)	9 (3.7)	82 (33.9)	41 (16.9)	4 (1.8)

**Table 4.** Resistance of the most frequently informed microorganisms recovered from the samples received at the hospital from the LTCF

Microorganism	Antibiotic	Percentage of resistance (%)
*Escherichia coli* (*n* = 38)	Ampicillin	94.7
	Amoxicillin/clavulanate	62.9
	Ciprofloxacin	83.8
	Cefuroxime	45.9
	Cotrimoxazol	78.4
	ESBL-producers	28.9
*Proteus mirabilis* (*n* = 17)	Ampicillin	70.6
	Amoxicillin/clavulanate	17.6
	Ciprofloxacin	82.4
	Cefuroxime	17.6
	Cotrimoxazol	41.2
*Staphylococcus aureus* (*n* = 24)	Cefoxitin	70.8
	Ciprofloxacin	91.7
	Levofloxacin	91.7
	Mupirocin	0.0
	Linezolid	87.5
*Klebsiella pneumoniae* (*n* = 38)	Amoxicillin/clavulanate	11.1
	Ciprofloxacin	44.4
	Cefuroxime	11.1
	Cotrimoxazol	44.4
	ESBL-producers	11.1
*Pseudomonas aeruginosa* (*n* = 11)	Ceftazidime	94.7
	Piperacillin-Tazobactam	62.9
	Ciprofloxacin	83.8
	Imipenem	45.9

Only the most usually prescribed antibiotics to the LTCF residents are shown.

**Table 5.** Antibiotic prescription during the years of the study in daily defined doses per 1.000 habitants per day (DID)

Antibiotic	DID
Amoxicillin/clavulanate oral	35.7
Levofloxacin oral	9.0
Cefuroxime axetil oral	7.6
Clarithromycin oral	4.6
Ciprofloxacin oral	4.1
Cotrimoxazol oral	3.6
Fosfomycin-trometamol	3.0
Amoxicillin/clavulanate intravenous	2.9
Azithromycin oral	2.5
Levofloxacin intravenous	2.4
Amoxicillin oral	2.1

### Clinical characteristics of participants who died during the study

**Figure 2.**
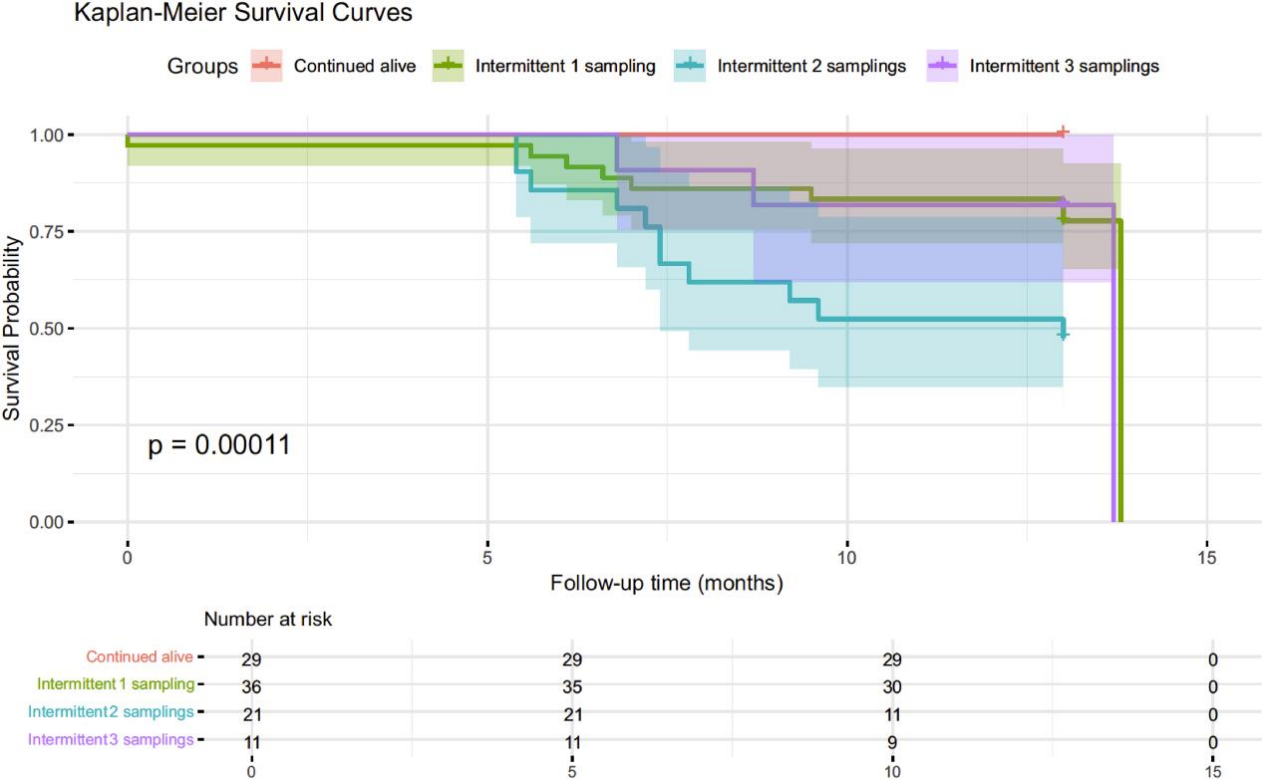
Kaplan–Meier analysis comparing the survival probability in the four groups of patients colonized by with of ESBL-producing *E. coli.*

The rate of mortality was comparable among colonized (23/97, 23.7%) and non-colonized participants (23/90, 25.5%). Both groups had suffered previous infectious, being more frequent in the colonized group, including four episodes by MDR microorganisms, thus requiring more hospital admissions and antimicrobial treatments (Table 6). Static differences in mortality were detected when comparing the four categories of colonized ESBL-producing *E. coli* residents: continuous colonization and intermittent colonization in one, two and three sampling sessions. A higher mortality rate was observed in the intermittent colonization group in two sampling sessions (11/21, 52%) compared to the rest (9/36, 25.0% in one colonization; and 3/11, 27.2% in three colonizations), especially with the continuous colonization group (Figure 2).

**Table 6.** Clinical data of the 46 residents who died during the study

Sampling	1	2	3	4	Total
Non-colonized	*n* = 23	*n* = 17	*n* = 15	*n* = 4	
Infections					
Respiratory	13	2	7	1	23
Urinary	4	2	1	0	7
Skin and soft tissues	2	1	1	0	4
MDR infections	0	0	0	0	0
Antibiotic therapy					
Amoxicillin/Clavulanate	6	2	4	0	12
Cephalosporin 3^a^	1	0	0	0	1
Fluoroquinolones	10	1	2	1	14
Fosfomycin	4	0	1	0	5
Aminoglycosides	1	0	0	0	1
Azithromycin	2	0	0	0	2
Cotrimoxazole	1	0	0	0	1
Hospital admissions	4	0	0	0	4
Surgical procedures	2	0	0	0	2
ESBL colonized	*n* = 23	*n* = 21	*n* = 13	*n* = 5	
Infections					
Respiratory	10	13	4	2	29
Urinary	7	5	3	1	16
Skin and soft tissues	2	3	3	0	8
MDR infections	2	2	0	0	4
Antibiotic therapy					
Amoxicillin/Clavulanate	2	6	2	0	10
Cephalosporin 3rd generation	2	1	1	0	4
Fluoroquinolones	7	1	2	2	12
Fosfomycin	2	3	2	1	8
Aminoglycosides	1	0	0	0	1
Azithromycin	1	0	1	0	2
Cotrimoxazole	2	3	0	0	5
Hospital admissions	3	3	3	0	9
Surgical procedures	4	1	0	0	5

It should be noted that at time of in the sampling five all were categorized as *exitus*.

## Discussion

Infectious diseases are frequent in the last stages of life, mainly affecting respiratory or urinary tracts and skin and soft tissues.^10,11^ While respiratory infections are often empirically treated without collecting any clinical sample, urine is the most frequent sample sent to the microbiology laboratory. It is important to note that asymptomatic bacteriuria is common in elderly, and but its management is often problematic as they do not usually present localized genitourinary symptoms. This situation is reflected by the fact that many urinary tract infections in elderly are overtreated, being this a controversial issue for the medical staff.^12^ In our centre, respiratory tract infections (40%) were the most common, followed by infections of the urinary tract (17%) and, to a lesser extent, those of skin and soft tissues (8%). The microorganisms’ distribution is comparable to those previously described in other studie.^10,13^ Isolates exhibited elevated rates of resistance to most antimicrobials, especially remarkable in the case of fluoroquinolones, particularly in *E. coli*, *P. mirabilis* and *P. aeruginosa*. It is important to highlight the percentage of carbapenem-resistant *P. aeruginosa* (8%) and MRSA (70%), with a lack of detection of carbapenemase-producing Enterobacteriaceae. During the year of the study, ESBL production rates in *E. coli* and *K. pneumoniae* were 28.9% and 11.1%, respectively, higher than expected and probably linked to the increase in oral ceftriaxone use. The LTCF medical staff is aware of the need for stewardship of antibiotics’ prescription, avoiding overtreatment. Many studies emphasize the disruptive effect of antibiotics in the integrity of elderly microbiome.^14^ Effectiveness of oral administration to avoid intravenous treatments has been evaluated in many clinical conditions and promoted when conclusive results were obtained.

The lack of gut colonization by *A. baumannii* and *C. difficile* as well as by carbapenemase-producing Enterobacterales was noticeable, even though these pathogens are common in residents of these institutions.^14–16^ A high prevalence of carbapenemase-producing isolates was detected colonizing elderly in various LTCTs in Spain.^8^ It has been established that antibiotics are the major selectors for MDR isolates,^17^ however, in our study, both colonized and non-colonized residents received similar dosages of antibiotics and have relatively similar infectious histories.

Along the year of our study, ESBL-producing *E. coli* colonized almost half of the residents, whereas *K. pneumoniae* was underrepresented, as has been previously reported.^18^ The prevalence of ESBL-producing *E. coli* in LTCF in other studies was considerably lower, 17% in Overdevest *et al*.,^19^ 22% in Kohler *et al*.^20^ and 18% in Martischang *et al.*.^21^

The expansion of MDR has been reported in the gut microbiota of people colonized by ESBL-producing *E. coli* when compared to those who are non-MDR bacteria carriers.^22^ This was probably in consonance with the dominance of specific well-adapted clones, such as ST131-H30 clone,^23^ which was also prevalent in our previous work.^7^ Controversially, in 2022, Ducarmon *et al*.,^24^ demonstrated the lack of alterations in the composition and functionality of gut microbiota in Dutch adults (not elderly) who were ESBL-*E. coli* carriers. The dynamics of gut colonization by MDR microorganisms in elderly attending LTCFs has been studied by many authors and results are not always coincident showing fluctuation rates even of prevalent clones. Tinelli *et al*.^16^ showed that 1 year after the beginning of their study, half of the participants had positive cultures. In 2021, Martischang *et al*.^21^ also described the clonal fluctuation of ESBL ST131 H30-producing *E. coli*, which also tended towards a progressive decolonization. Some individuals seem to be resistant to external colonization, as described by Ludden *et al*.,^25^ with a proportion of 40% of never-colonized residents.

Our initial hypothesis was that there would be a high mortality rate among ESBL-producing carriers, particularly due to infections caused by these bacteria. However, the group with the highest mortality was the group with two intermittent colonizations in which half of the participants died, whereas the mortality rates for residents with colonization in one or three samplings were 25.9% and 27.2%, respectively. Interestingly, we found no differences in antibiotic prescription between the groups, which cannot be justified by selection after antibiotic therapy. The explanation for this phenomenon is not simple, and is probably related to the stability of the gut microenvironment, which limits the expansion of MDR bacteria population and prevents systemic infections.

Our results are valid for our institution and cannot be extrapolated to other LTCFs. In fact, the screening of gut microbiome status of residents at their LTCF admittance could be an effective measured for MDR control, as well as serial sampling sessions to prevent the dispersion of MDR clones. Also minimizing both hospital admissions and antibiotic exposition is strongly promoted, as has been published in the ECDC recommendations.^26^ The main limitation of this study is that just one centre was evaluated for only 1 year. More extensive studies will probably be necessary to fully understand the dynamics of colonization/decolonization in relation to infections and antibiotic exposure. However, the huge collection of clinical data that allowed us to assign a mortality risk in elderly subjects colonized by MDR microorganisms is certainly valuable. The lack of characterization of the microbiota of the residents, and genetic comparison between MDR-colonizing and infecting bacteria are major limitations of our work.

In conclusion, the monitorization of the gut colonization by MDR microorganisms in a single LTCF during 1 year through five sampling sessions by rectal swabs demonstrated the predominance of ESBL-*E. coli* with a lack of other pathogens. We detected residents who were never colonized, others who were always colonized and those who were intermittently colonized. In these three categories, the infection episodes and the antibiotic exposure were comparable. However, the group with the highest risk of mortality were those intermittently colonized, specifically those who presented two out of five positive samples.
